# Uncovering the Mechanisms of Cryptotanshinone as a Therapeutic Agent Against Hepatocellular Carcinoma

**DOI:** 10.3389/fphar.2020.01264

**Published:** 2020-08-13

**Authors:** Yi Luo, Lei Song, Xinyu Wang, Yujie Huang, Yongqiang Liu, Qi Wang, Ming Hong, Zhongyu Yuan

**Affiliations:** ^1^Science and Technology Innovation Center, Guangzhou University of Chinese Medicine, Guangzhou, China; ^2^Institute of Clinical Pharmacology, Guangzhou University of Chinese Medicine, Guangzhou, China; ^3^Department of Medical Oncology, Sun Yat‐sen University Cancer Center, Guangzhou, China

**Keywords:** hepatocellular carcinoma, cryptotanshinone, system pharmacology, apoptosis, autophagy

## Abstract

Hepatocellular carcinoma (HCC) is a fatal and dominant form of liver cancer that currently has no effective treatment or positive prognosis. In this study, we explored the antitumor effects of cryptotanshinone (CPT) against HCC and the molecular mechanisms underlying these effects using a systems pharmacology and experimental validation approach. First, we identified a total of 296 CPT targets, 239 of which were also HCC-related targets. We elucidated the mechanisms by which CPT affects HCC through multiple network analysis, including CPT-target network analysis, protein-protein interaction network analysis, target-function network analysis, and pathway enrichment analysis. In addition, we found that CPT induced apoptosis in Huh7 and MHCC97-H ells due to increased levels of cleaved PARP, Bax, and cleaved caspase-3 and decreased Bcl-2 expression. CPT also induced autophagy in HCC cells by increasing LC3-II conversion and the expression of Beclin1 and ATG5, while decreasing the expression of p62/SQSTM1. Autophagy inhibitors (3-methyladenine and chloroquine) enhanced CPT-induced proliferation and apoptosis, suggesting that CPT-induced autophagy may protect HCC cells against cell death. Furthermore, CPT was found to inhibit the phosphatidylinositol 3-kinase (PI3K)/protein kinase B (Akt)/mammalian target of rapamycin (mTOR) signaling pathway. Interestingly, activation of PI3K by insulin-like growth factor-I inhibited CPT-induced apoptosis and autophagy, suggesting that the PI3K/AKT/mTOR signaling pathway is involved in both CPT-induced apoptosis and autophagy. Finally, CPT was found to inhibit the growth of Huh7 xenograft tumors. In conclusion, we first demonstrated the antitumor effects of CPT in Huh7 and MHCC97-H cells, both *in vitro* and *in vivo*. We elucidated the potential antitumor mechanism of CPT, which involved inducing apoptosis and autophagy by inhibiting the PI3K/Akt/mTOR signaling pathway. Our findings may provide valuable insights into the clinical application of CPT, serving as a potential candidate therapeutic agent for HCC treatment.

## Introduction

Hepatocellular carcinoma (HCC) is a type of primary liver malignancy that typically occurs in the context of chronic liver inflammation and is the most common cause of cancer-related deaths worldwide (Forner et al., 2012). The clinical characteristics of HCC include abdominal pain, weight loss, and a large mass in the upper right part of the abdomen. While the etiology of HCC is not fully elucidated, there is a consensus that the main risk factors of HCC seem to be associated with hepatitis B and C virus infection, obesity, diabetes, alcohol intake, or exposure to aflatoxin B1 (Duran and Jaquiss, 2019). Currently, HCC treatment includes surgical resection and comprehensive treatment (immunotherapy, radiotherapy, chemotherapy, interventional therapy, or a combination); however, the overall survival rate of patients with HCC remains low. Liver transplantation, partial ablation, and liver resection remain the mainstream curative options for very early and early-stage HCC HCC (Montironi et al., 2019; Sarcognato et al., 2019). However, most patients are typically diagnosed at an advanced stage with severe comorbidities, making them unsuitable for surgery. At more advanced stages, the overall survival rate can be improved by sorafenib treatment, but this is associated with significant side effects and potential toxicity (Ayoub et al., 2019; Inchingolo et al., 2019; Lu et al., 2019). As such, there is an urgent need to develop novel HCC therapeutic strategies.

Cryptotanshinone (CPT), an active compound isolated from Radix *Salviae Miltiorrhizae*, has been reported to possess diverse pharmacological activities, including anti-fibrosis, neuroprotective, anti-inflammatory, anti-atherosclerotic, and antioxidant activities (Mei et al., 2009; Wong et al., 2010; Ran et al., 2016; Lo et al., 2017; Bai et al., 2019; Sun et al., 2019; Zhou et al., 2019). For example, CPT reduced inflammation and oxidative stress in renal interstitial fibrosis by modulating the Nrf-2/HO-1 and NF-kappaB signaling pathways (Wang et al., 2018). Additionally, CPT has been reported to exert anticancer effects on multiple types of cancers, including osteosarcoma, lung cancer, esophageal squamous cell carcinoma, colon cancer, ovarian cancer, chronic myeloid leukemia, gastric cancer, breast cancer, renal cell carcinoma, and melanoma (Chen et al., 2017; Pan et al., 2017; Wang et al., 2017a; Dong et al., 2018; Yang et al., 2018; Zhang et al., 2018b; Ji et al., 2019). In lung cancer cells, CPT exhibits antitumor effects by inhibiting cell proliferation and migration through inhibition of insulin-like growth factor-I (IGF-1)R-mediated phosphatidylinositol 3-kinase (PI3K)/protein kinase B (Akt) signaling pathway (Zhang et al., 2018a). Additionally, CPT has also been shown to inhibit cell proliferation and exert immunotherapeutic effects in Lewis lung carcinoma (Liu et al., 2019). Moreover, CPT has been shown to play antitumor roles by inhibiting proliferation and inducing apoptosis human chronic myeloid leukemia cells through the eIF4E regulatory system (Ge et al., 2012). Recently, two studies have demonstrated that CPT is capable of inhibiting tumor growth in HCC. One study reported that CPT exhibits antitumor effects against HCC and induces antitumor immunity *in vivo* and *in vitro* (Han et al., 2019). Another study reported that CPT inhibits prostaglandin E2-induced apoptosis and invasion of HA22T HCC cells *via* the β-catenin pathway (Chang et al., 2018). However, the mechanisms by which CPT effectively treats HCC *in vitro* and *in vivo* are still not fully elucidated.

**Figure 1:**
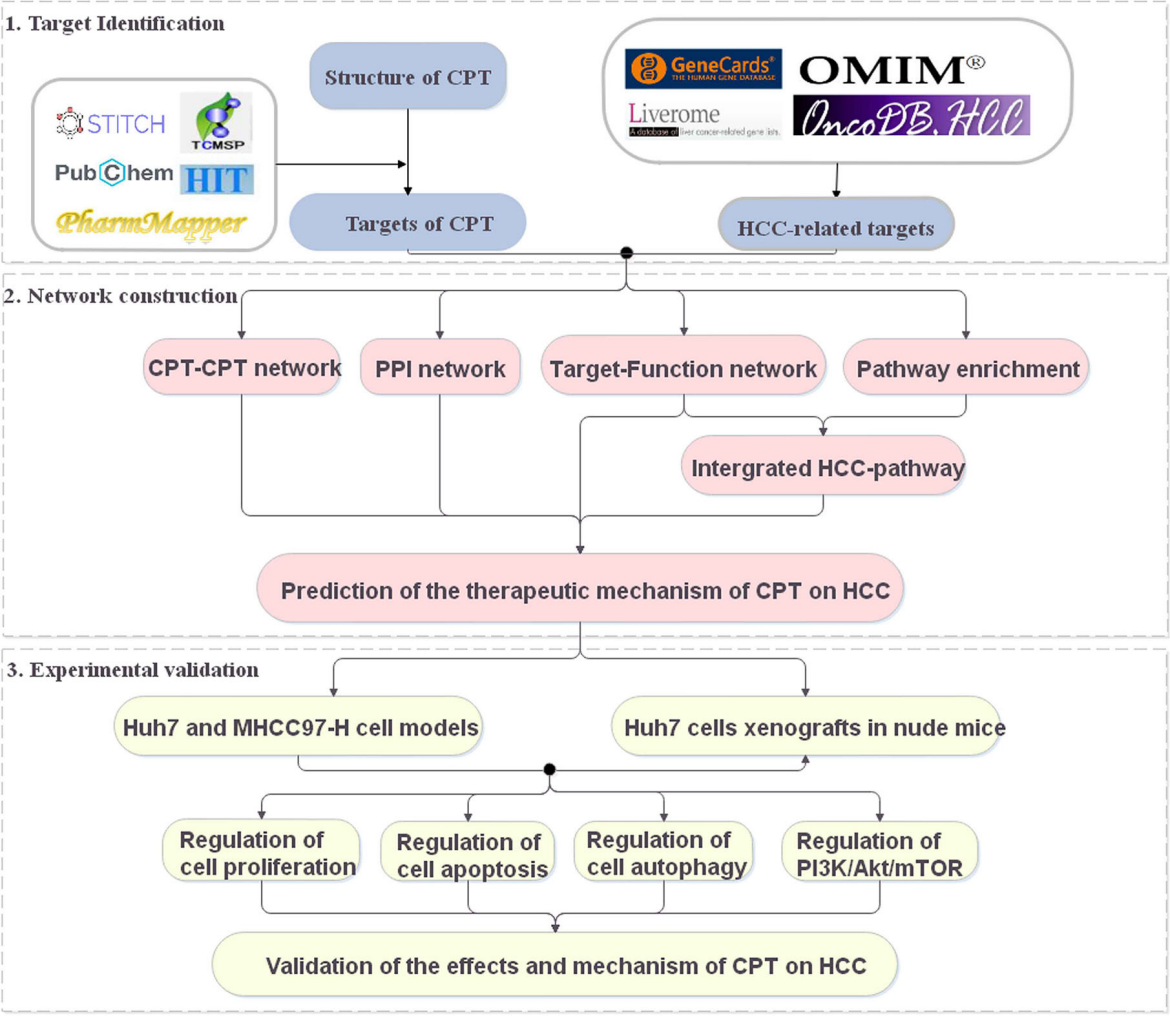
A schematic diagram showing the systems pharmacology approach for investigating the therapeutic mechanisms of cryptotanshinone (CPT) on human hepatocellular carcinoma (HCC) by integrating target identification, network analysis, and experimental validation.

Systems pharmacology is an emerging discipline investigating the interactions between drugs and the body and their underlying rules and mechanisms of action from a systems-level perspective (Huang et al., 2014; Chen et al., 2016). In recent years, systems pharmacology has been used to identify active compounds and medicinal ingredients in Chinese medicine and has provided new ideas and perspectives for the study of complex Chinese medicine systems (Fang et al., 2017). In this study, a systems pharmacology approach was used to explore the potential targets and therapeutic mechanisms of CPT in HCC treatment (**Figure 1**). The potential targets of CPT were mapped to HCC-related databases to explore their biological functions and associated HCC pathways. Using the above results, we constructed networks to investigate the effects and mechanisms of CPT action on HCC. Finally, we used *in vitro* and *in vivo* experiments to validate the proposed effects and mechanisms of CPT as an anti-HCC agent.

## Materials and Methods

### Collection of Potential Targets for HCC

Potential targets of CPT were collected by using five databases, including Traditional Chinese Medicine Systems Pharmacology (TCMSP) (Ru et al., 2014) (http://tcmspw.com/tcmsp.php; Updated:2014), Pubchem (Kim et al., 2019) (https://pubchem.ncbi.nlm.nih.gov; Updated:2019), STITCH (Kuhn et al., 2014) (http://stitch.embl.de/; Version:5.0), HIT (Ye et al., 2011) (http://lifecenter.cn/hit/; Update:2011) and PharmMapper (Wang et al., 2017b) (http://www.lilab-ecust.cn/pharmmapper/; Updated:2017).

### HCC-Related Targets

To construct the disease target database, we collected HCC-related targets from four databases: GeneCards (Stelzer et al., 2016) (http://www.genecards.org; Version: 4.12.0), Online Mendelian Inheritance in Man (OMIM) (Amberger and Hamosh, 2017) (https://omim.org; Updated: 2019), Liverome (Lee et al., 2011) (http://liverome.kobic.re.kr/; Updated: 2011), and OncoDB.HCC (Su et al., 2007) (http://oncodb.hcc.ibms.sinica.edu.tw/index.htm; Updated: 2008).

### Protein-Protein Interaction (PPI) Data

PPI data were obtained from a free biological database known as Search Tool for the Retrieval of Interacting Genes/Proteins (STRING) (Szklarczyk et al., 2015) (http://string-db.org/; version 11.0), with the species limited to “*Homo sapiens*.”

### Gene Ontology (GO) and Pathway Enrichment Analysis

Database for Annotation, Visualization and Integrated Discovery (Huang et al., 2009) (DAVID; http://david.abcc.ncifcrf.gov/; Version 6.8) was used to enrich GO and pathway enrichment analysis.

### Network Construction

To further explore the therapeutic mechanisms of the CPT in HCC, we constructed the CPT-CPT target network, PPI network and Target–Function network. All visualized networks were generated using the software Cytoscape (Shannon et al., 2003) (version 3.7.2).

### Reagents and Antibodies

CPT was purchased from Sigma-Aldrich (≥98% purity; St. Louis, MO, USA) and dissolved in dimethyl sulfoxide (DMSO) as a stock solution at 0.1 M. Chloroquine (CQ) and 3-Methyladenine (3-MA) were purchased from Sigma-Aldrich. Insulin-like growth factor-I (IGF-I) was obtained from R&D Systems, Inc. (Minneapolis, MN, USA). Annexin V-FITC/PI Apoptosis Detection Kit was purchased from Vazyme (Nanjing, China). BeyoClick™ EdU-555 Cell Proliferation Assay Kit was purchased from Beyotime (Shanghai, China). Antibodies, including PI3-kinase p-p85-α (ab182651), Bcl-2 (ab59348), HRP-conjugated goat anti-rabbit (ab6721), and anti-mouse IgG (ab6789) were purchased from Abcam Co. (Cambridge, MA, USA). Antibodies, including AKT (#9272), p-AKT (#4058), Bax (#2772), PARP (#9532), Caspase-3 (#9662), Cleaved caspase-3(#9664), LC3B (#3868), Beclin-1 (#3495), SQSTM1/p62 (#8025), and β-actin (ab8227) were obtained from Cell Signaling Technology Inc. Antibodies, including anti-PI3-kinase p85-α (SAB4502195), were purchased from Sigma-Aldrich.

### Cell Lines and Culture

HCC cell lines Huh7 and MHCC97-H were provided by the Cell Bank of Type Culture Collection of the Chinese Academy of Sciences (Shanghai, China). All HCC cell lines were maintained in Dulbecco’s Modified Eagle’s Medium (DMEM; Gibco, Grand Island, NY, USA) supplemented with 10% fetal bovine serum (FBS; Gibco) and 100 U/ml penicillin-streptomycin (Gibco) at 37°C in a humidified incubator with 5% CO_2_.

### 3-(4,5-Dimethylthiazol-2-yl)-2,5-Diphenyltetrazolium Bromide (MTT) Assay

Cell viability was evaluated using the MTT assay. Huh7 and MHCC97-H cells were seeded in 96-well plates (8×10^3^/well) and maintained in DMEM medium overnight. A volume of 10 μl of MTT (5 mg/ml) solution was added to each well at specific time points and incubated at 37°C for 4 h. After the cultured medium was removed, the crystals were dissolved in 150 μl of DMSO, and the absorbence was detected using a microplate reader (Thermo Fisher, USA) at 490 nm.

### Colony Formation Assay

Cell proliferation ability was assessed using the colony formation assay. Huh7 and MHCC97-H cells were seeded in 6-well plates (10^3^/well), cultured in different reagents, as indicated, and then maintained in DMEM medium with 10% FBS. After 14 days, colonies were stained with crystal violet for 10 min before being fixed with 4% paraformaldehyde for 10 min. Cell colonies (>50 cells/colony) were counted.

### EdU Assay

To evaluated cell proliferation ability, Huh7 and MHCC97-H cells were detected using the BeyoClick™ EdU-555 Assay, according to the manufacturer’s instructions (Beyotime, Guangzhou, China). Fluorescence microscopy was used to photograph the cells, and the fluorescence of the EdU-positive cells was measured using Image-pro Plus 6.0 (NIH, Bethesda, MD, USA).

### Flow Cytometry for Cell Apoptosis Analysis

An annexin V-FITC apoptosis detection kit was used to assess apoptosis. Briefly, Huh7 and MHCC97-H cells were treated with various drugs and then stained using the annexin V-FITC apoptosis detection kit, according to the manufacturer’s protocol. Apoptotic cells were detected and analyzed by flow cytometry (BD Biosciences, USA).

### TUNEL Assays

Tumor cells or tissue sections were used for terminal deoxynucleotidyl transferase-mediated dUTP nick-end labeling (TUNEL) assays to detect apoptosis using a TUNEL apoptosis assay kit acquired from Beyotime, according to the manufacturer’s protocol. TUNEL-positive cells were imaged under a fluorescence microscope.

### Transmission Electron Microscopy (TEM)

Huh7 and MHCC97-H cells were fixed with 2.5% glutaraldehyde in 0.1 M sodium cacodylate. After fixation, samples were embedded using 1% osmium tetroxide and dehydration. Uranyl acetate and lead citrate (3%) were used to stain the ultrathin sections. Images were acquired with a JEM-1200 electron microscope (JEOL, Tokyo, Japan).

### Immunofluorescence

Huh7 and MHCC97-H cells were grown on glass coverslips and fixed with 4% paraformaldehyde for 20 min, permeabilized in 0.2% Triton X-100, and blocked with 5% bovine serum albumin (BSA) for 30 min. Cells were stained with primary antibody and subsequently incubated with secondary antibodies at 37°C for 1 h. Finally, cells were stained with 4′,6-diamidino-2-phenylindole (DAPI) (Beyotime). Cells were imaged using a Zeiss LSM 710 (Carl Zeiss Microscopy GmbH, Jena, Germany) laser scanning confocal microscope.

### Western Blotting

Total cell or tissue proteins were extracted with lysis solution. Protein extracts were subjected to sodium dodecyl sulfate-polyacrylamide gel electrophoresis (SDS-PAGE) and then transferred onto polyvinylidene difluoride (PVDF) membranes (Bio-Rad, Hercules, CA). Membranes were blocked for 1 hour with 5% BSA at room temperature and then incubated with primary antibodies overnight at 4°C. After washing, the membranes were probed with goat anti-rabbit or goat anti-mouse HRP-conjugated secondary antibodies for 1 hour at room temperature. Finally, after the membranes were washed three times, protein bands were detected using an enhanced chemiluminescence kit (ECL Kit; Pierce Biotech, Rockford, IL, USA). The information on all antibodies is listed in the *Materials and Methods* section.

### Molecular Docking Studies

Molecular docking simulations were used to explore the potential interaction between CPT and PI3K. The three-dimensional (3D) crystal structure of PI3K (PDB code: 5ITD) was prepared using Autodock 4.2.6 (http://autodock.scripps.edu/) for docking studies. The 3D structure of CPT (Pubchem CID: 160254) was modeled with energy minimized using ChemOffice software (CambridgeSoft, Cambridge, MA, USA). To perform docking simulations in Autodock 4.2.6, the grid dimensions were established using the grid center, spacing (1.0 Å), and the number of points (X: 98, Y: 60, Z: 90). Molecular docking simulations were performed and analyzed using a Lamarckian genetic algorithm method (Runs 100) implemented using Autodock Vina 1.1.2.

### Tumor Xenograft in Nude Mice

Four-week-old male BALB/c nude mice were purchased from the Experimental Animal Center of Guangzhou University of Traditional Chinese Medicine, China. Huh7 cells (5×10^6^/100 μl) were injected subcutaneously into the axilla of each mouse to establish the HCC xenograft model. Five days after subcutaneous inoculation, mice were randomly assigned to two groups: a control group (n = 6, 0.9% normal saline, 0.1 ml/10g) and a CPT-treated group (n = 6, 50 mg/kg, 0.1 ml/10g). CPT was prepared with a saline solution containing 5% (v/v) N’, N-dimethylacetamide and 5% (v/v) polyoxyl 15 hydroxystearate and administered *via* intraperitoneal injection every day. The length and width of the tumors (in millimeters) were measured every two days using calipers. Tumor volume was estimated using the formula (L×W^2^)/2, where L and W are the tumor length and width, respectively. After 21 days, the nude mice were sacrificed, and the tumors were extracted and weighed. Mice experiments were performed by the National Institutes of Health Guidelines for the Care and Use of Laboratory Animals. The study protocols were approved by the Institutional Animal Care and Use Committee of Guangzhou University of Traditional Chinese medicine, China.

### Statistical Analysis

Data were analyzed using the SPSS20.0 software package (Chicago, IL, USA). Student’s t-test and one-way analysis of variance, followed by Dunnett’s *post hoc* test were conducted. Data are shown as the mean ± standard deviation (SD). *P* < 0.05 was considered statistically significant.

## Results

### Construction of the CPT-CPT Target Network

**Figure 2:**
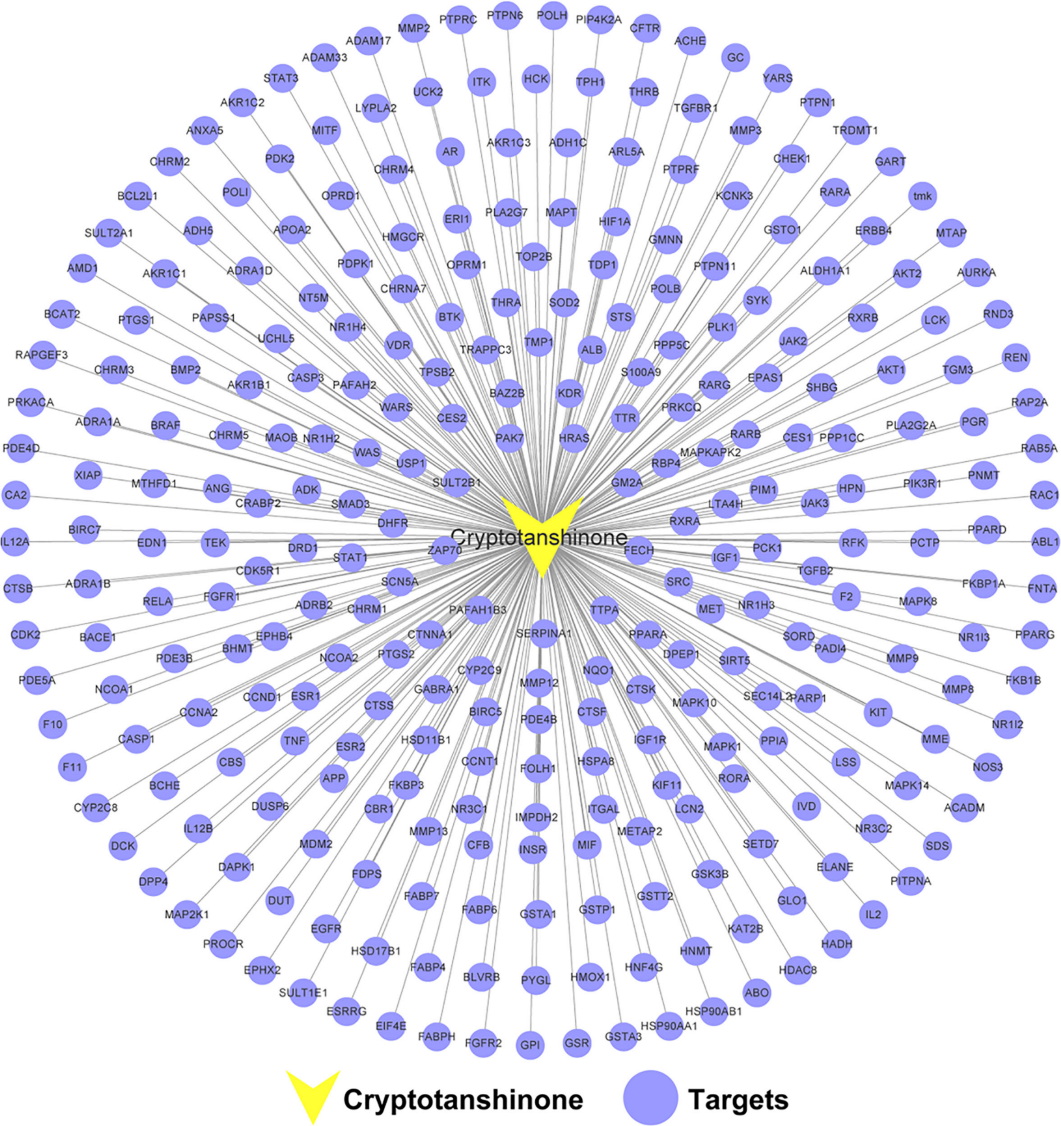
Cryptotanshinone (CPT)-CPT target network. The network includes CPT and 296 CPT targets.

A total of 296 targets were obtained for further analysis after the removal of duplicate targets. Details are provided in **Supplementary Table S1**. To elucidate the interactions between CPT and CPT targets, we generated a CPT-CPT target network. As shown in **Figure 2**, the CPT-CPT target network consisted of 297 nodes (1 compound and 296 targets) and 296 CPT-CPT target interactions. The results of the CPT-CPT target network analysis reveal that CPT has multiple targets, including AKT1, PIK3R1, CASP3, GSK3B, and HSP90AA1. In conclusion, the multi-target characteristic of CPT prompted us to elucidate the mechanism of CPT action against HCC through systems pharmacology analysis of the interactions between CPT and these 296 targets.

### Identification of Targets for CPT Acting on HCC

To construct the HCC-related targets database, we respectively collected 609 targets from Liverome, 6,927 targets from OncoDB. HCC, 5,290 targets from GeneCards, and 299 targets from OMIM. The details are shown in **Supplementary Table S2**. To further explore the efficacy and mechanisms of CPT action on HCC, we conducted PPI network analysis, Target–Function network analysis, and pathway enrichment analysis on the shared targets of CPT and HCC. Detailed information regarding the common targets of CPT and HCC is shown in **Supplementary Table S3**.

### PPI Network Analysis

**Figure 3:**
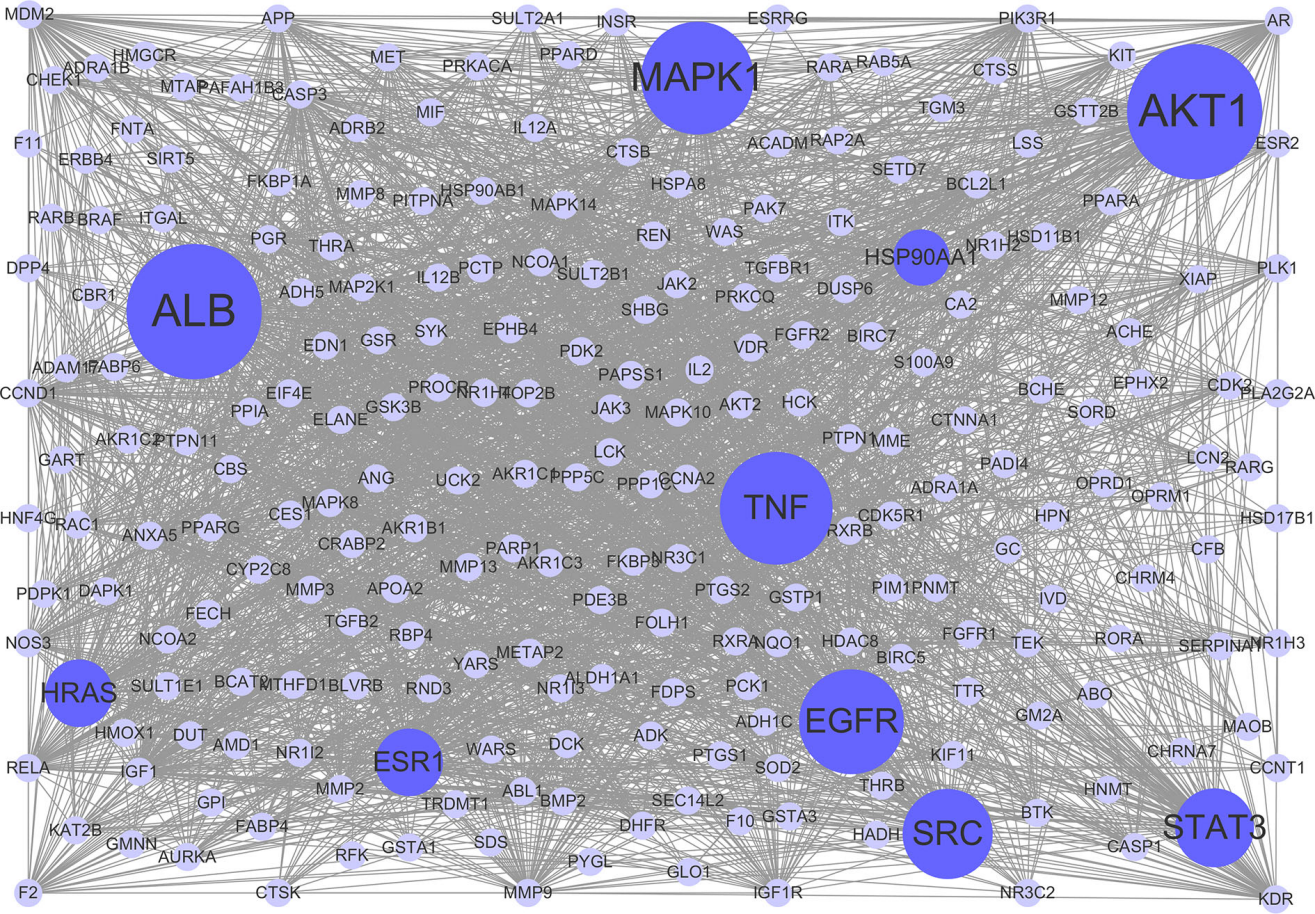
Protein-protein interaction (PPI) network. The nodes get larger with increasing degree. Edges: PPIs between targets of cryptotanshinone (CPT) and their interaction partners; purple circle nodes: common targets of CPT and human hepatocellular carcinoma (HCC); dark purple circle nodes: hub targets of CPT and HCC (Degree ≥ 80).

To clarify the interactions between common targets, a PPI network was constructed. As shown in **Figure 3**, this network consisted of 237 nodes and 2,768 edges. The average degree node of the common targets was 23.36. In total, 32 hub targets were identified, whose node degrees were two-fold greater than the average node degree in this network. The nodes interacted with others *via* numerous edges (115 in AKT1, 115 in ALB, 94 in MAPK1, 94 in TNF, 93 in EGFR, 90 in SRC, 86 in STAT3, 83 in HRAS, 83 in ESR1, and 81 in HSP90AA1). The results of this network suggest that these hub targets may play a crucial role in the treatment of HCC with CPT. Detailed information on the 32 hub targets in this PPI network is presented in **Supplementary Table S4**.

### Target–Function Network Analysis

**Figure 4:**
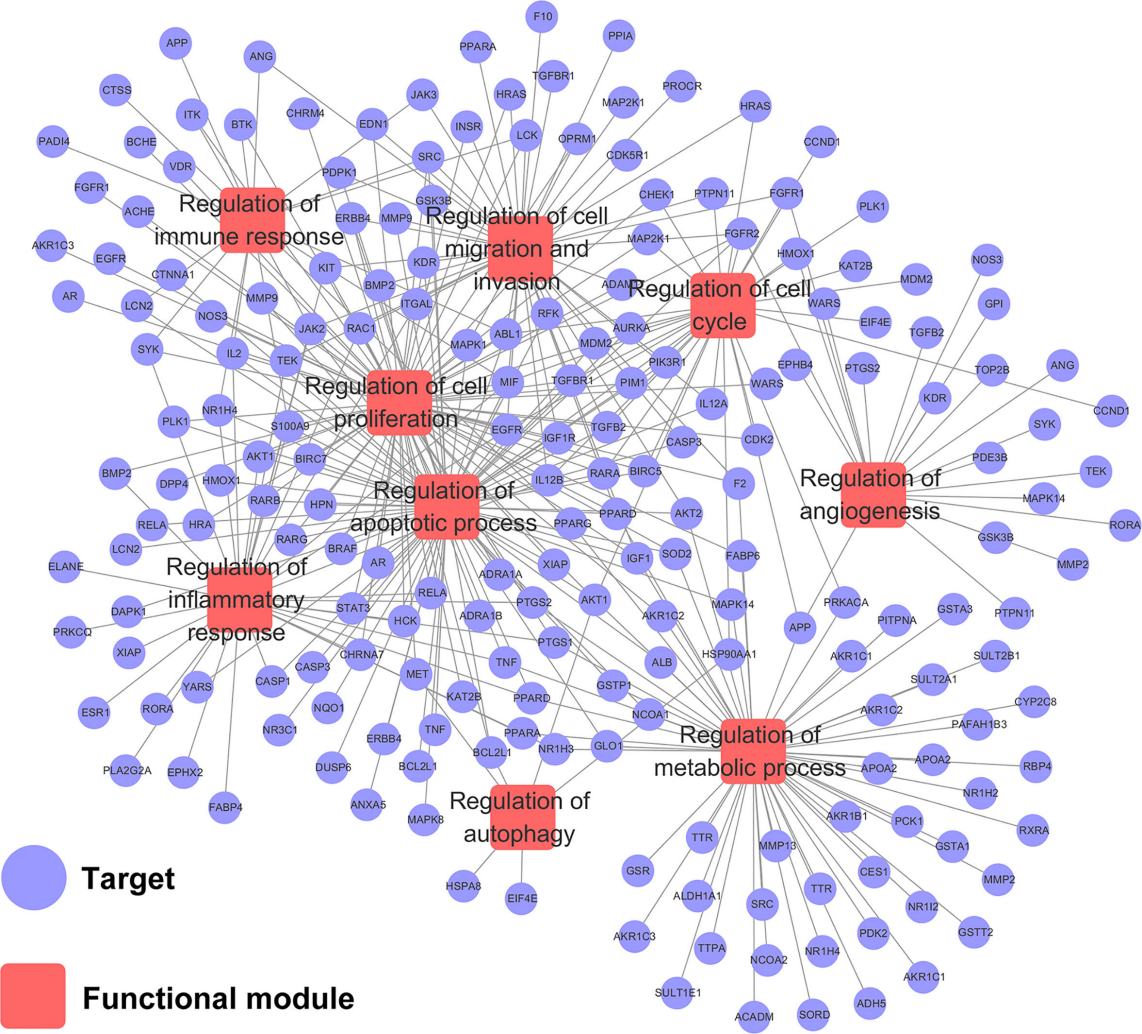
Target-function network. The functional module is connected to the target when the target participates in that biological process.

To explore the relationships between HCC-related biological processes and associated targets, we constructed a Target–Function network. As shown in **Figure 4**, this network included 359 target-function pairs connecting 202 targets with 9 HCC-related functional modules obtained using DAVID analysis (**Supplementary Table S5**). The nine functional modules included regulation of angiogenesis, autophagy, immune response, inflammatory response, cell proliferation, cell cycle, apoptotic process, cell migration and invasion, and metabolic processes. Previous studies have validated that CPT is able to inhibit cell proliferation and induce apoptosis, cell cycle arrest, immune defense, and autophagic cell death. For instance, CPT induces G1 cell cycle arrest and autophagic cell death *via* activation of AMPK signaling pathway (Park et al., 2014). However, there is no literature exploring the relationship between apoptosis and autophagy. Therefore, we will explore the relationship between CPT-induced apoptosis and autophagy in Huh7 and MHCC97-H cells in future studies.

### Pathway Enrichment Analysis for Targets

**Figure 5:**
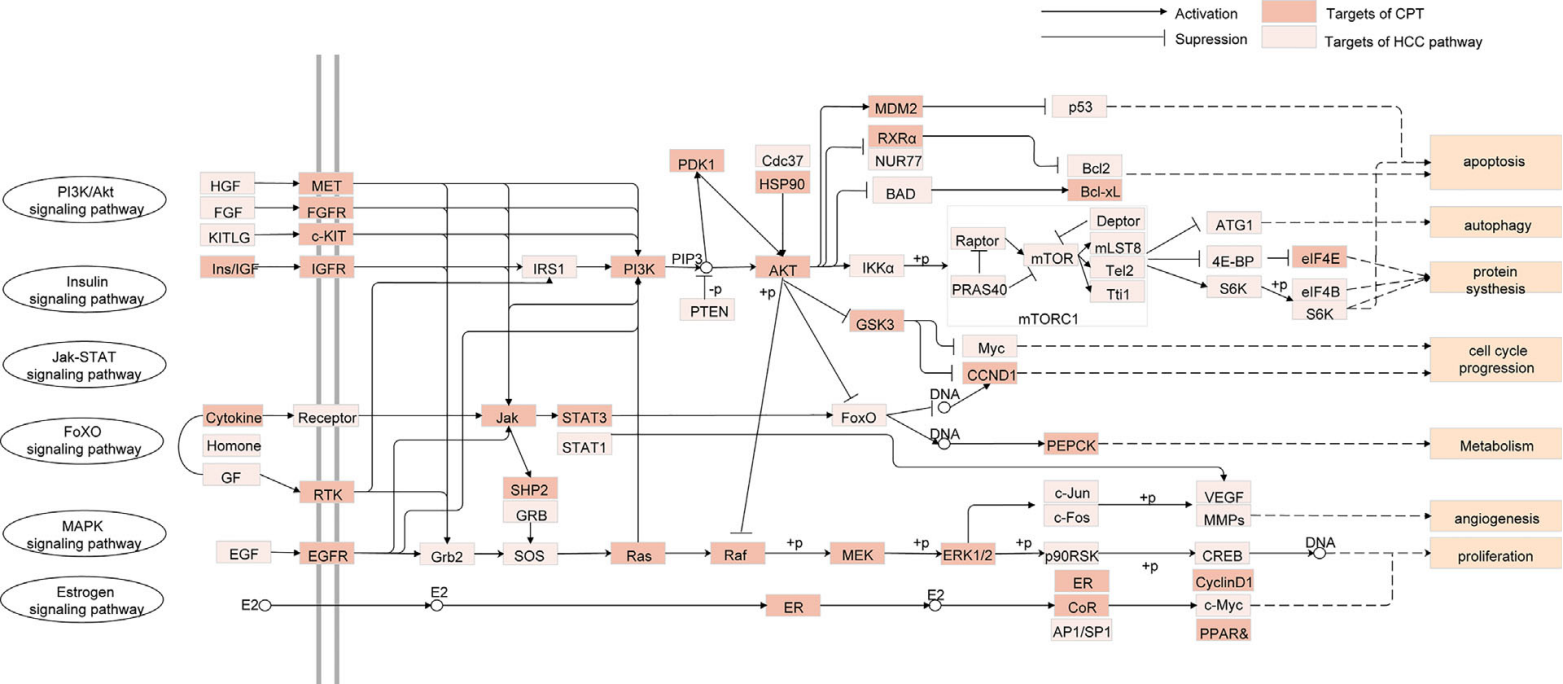
Integrated human hepatocellular carcinoma (HCC)-pathway and functional modules.

To investigate the therapeutic mechanisms of CPT in HCC treatment, we conducted pathway enrichment analysis of the common targets of CPT and HCC using DAVID. Detailed pathway information is shown in **Supplementary Table S6**. The pathways correlated with HCC were integrated into an “HCC-pathway” network based on HCC pathogenesis target prediction. The mechanisms involved in treating HCC with CPT may be closely associated with these pathways, such as the FoxO signaling pathway, PI3K-Akt signaling pathway, and the estrogen signaling pathway, as shown in **Figure 5**. The PI3K/Akt signaling pathway is involved in the regulation of a variety of biological processes in normal cells, including cell proliferation, apoptosis, survival, growth, and movement, all of which are closely associated with tumorigenesis. Therefore, we further validated whether PI3K/Akt signaling pathway is involved in the treatment of HCC with CPT.

### CPT Inhibits the Proliferation of Huh7 and MHCC97-H Cells

**Figure 6:**
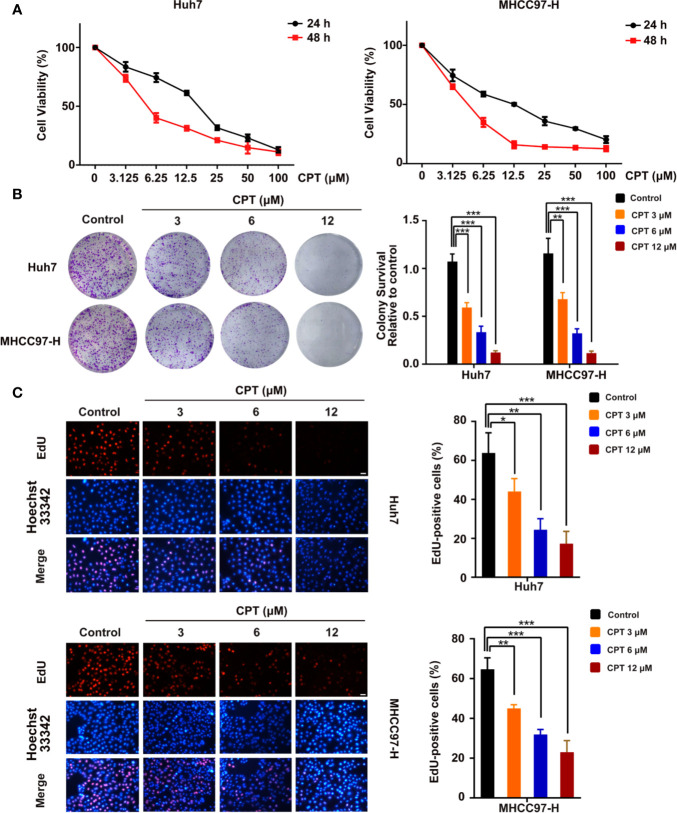
Cryptotanshinone (CPT) inhibits the proliferation of Huh7 and MHCC97-H cells. **(A)** 3-(4,5-dimethylthiazol-2-yl)-2,5-diphenyltetrazolium bromide (MTT) analysis of human hepatocellular carcinoma (HCC) cell lines, including Huh7 and MHCC97-H cells, treated with the indicated doses of CPT for 24 and 48 h. These experiments were independently repeated three times. **(B)** A colony formation assay was performed for Huh7 and MHCC97-H cells incubated with various doses (0, 3, 6, and 12 μM) of CPT. Colony survival relative to control groups was calculated and presented as mean ± standard deviation (SD) (n = 3). **(C)** Proliferative capacity was measured by EdU assay in Huh7 and MHCC97-H cells treated with the indicated concentrations of CPT (0, 3, 6, and 12 μM) for 24 h. *P < 0.05, **P < 0.01, and ***P < 0.001 versus the control group.

To assess the viability and anti-proliferative effects of CPT, MTT, colony formation, and EdU assays were performed on Huh7 and MHCC97-H cells. As shown in **Figure 6A**, CPT remarkably inhibited the viability of Huh7 and MHCC97-H cells upon treatment for 24 and 48 h. As shown in **Figure 6B**, CPT suppressed colony formation in both Huh7 and MHCC97-H cells at the indicated concentrations. The EdU assay further revealed that CPT decreased the percentage of EdU-positive cells in a dose-dependent manner (**Figure 6C**). These findings clearly suggest that CPT has anti-proliferative effects against Huh7 and MHCC97-H cells.

### CPT Induces Apoptosis of Huh7 and MHCC97-H Cells

**Figure 7:**
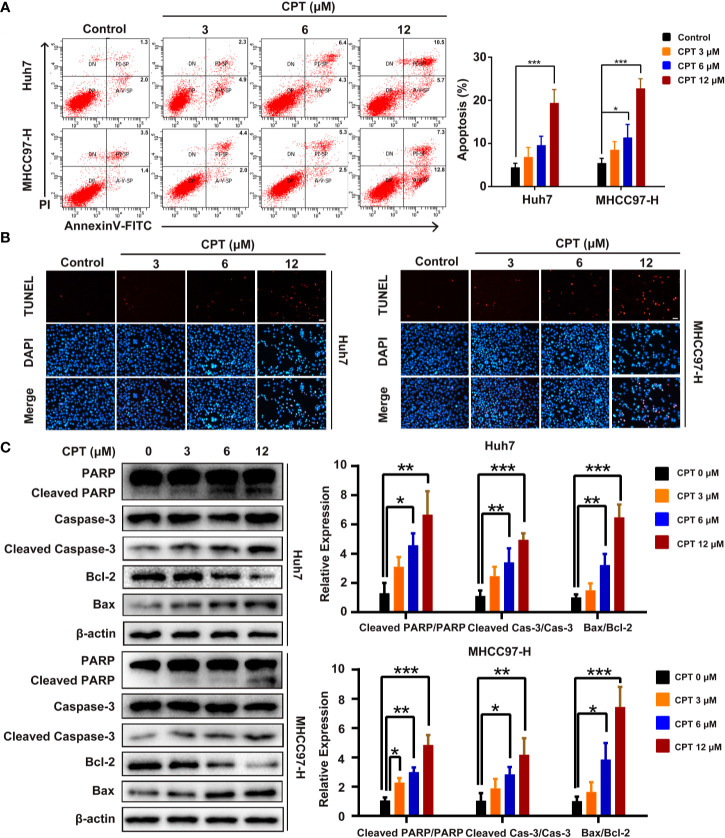
Cryptotanshinone (CPT) induces apoptosis in Huh7 and MHCC97-H cells. **(A)** Huh7 and MHCC97-H cells were treated with CPT (0, 3, 6, and 12 μM) for 24 h and analyzed using the Annexin V-FITC apoptosis detection kit. **(B)** TUNEL assay in Huh7 and MHCC97-H cells after treatment with CPT (0, 3, 6, and 12 μM). **(C)** Expression levels of PARP, cleaved PARP, caspase-3, cleaved caspase-3, Bcl-2, and Bax proteins in Huh7 and MHCC97-H cells following CPT (0, 3, 6, and 12 μM) treatment were detected by western blotting. *P < 0.05, **P < 0.01, and ***P < 0.001 versus the control group.

We next explored whether the effect of CPT on Huh7 and MHCC97-H cells is caused by apoptosis. As shown in **Figure 7A**, CPT treatment for 24 h increased the percentage of apoptotic cells in a dose-dependent manner. In the TUNEL assay, there was a significant increase in the number of apoptotic Huh7 and MHCC97-H cells after CPT treatment for 24 h (**Figure 7B**). In addition, we also examined the expression levels of apoptosis-related proteins, including PARP, cleaved PARP, caspase-3, cleaved caspase-3, Bcl-2, and Bax by western blotting. Results indicated that CPT treatment for 24 h decreased the expression level of Bcl-2 and increased the expression levels of cleaved PARP, cleaved caspase-3, and Bax (**Figure 7C**). Collectively, these findings indicate that CPT treatment induces apoptosis in Huh7 and MHCC97-H cells.

### CPT Stimulates Autophagy in Huh7 and MHCC97-H Cells

**Figure 8:**
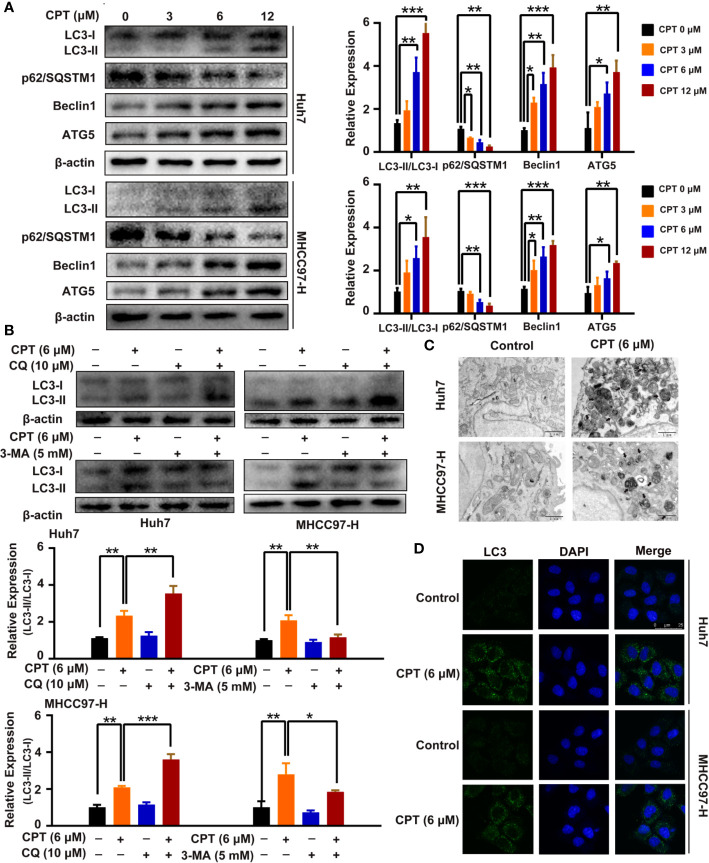
Cryptotanshinone (CPT) stimulates autophagy in Huh7 and MHCC97-H cells. **(A)** Western blot analysis of LC3-I, LC3-II, p62/SQSTM1, Beclin1, and ATG5 in human hepatocellular carcinoma (HCC) cells treated with the indicated concentrations of CPT (0, 3, 6, and 12 μM) for 24 h. *P < 0.05, **P < 0.01, and ***P < 0.001 versus the control group. **(B)** Western blot analysis of LC3-I conversion to LC3-II in Huh7 and MHCC97-H cells treated with 6 μM CPT in the absence or presence of 10 μM chloroquine (CQ) or 5 mM 3-methylamphetamine (3-MA) for 24 h was conducted. *P < 0.05, **P < 0.01, and ***P < 0.001 versus the CPT group. **(C)** Huh7 and MHCC97-H cells were treated with or without 6 μM CPT for 24 h. LC3B puncta were then stained with anti-LC3B antibody by immunofluorescence and imaged with a confocal microscope. **(D)** Cells were treated with or without 6 μM CPT for 24 h and the changes in ultrastructure were detected by transmission electron microscopy. Arrows: autophagosomes/autolysosomes. Scale bar: 1 µm.

A growing body of evidence has highlighted the important role of autophagy in anticancer therapy; therefore, we explored whether CPT regulates autophagy in Huh7 and MHCC97-H cells. CPT was found to induce the conversion of LC3-I to LC3-II in a dose-dependent manner in Huh7 and MHCC97-H cells. In addition, CPT downregulated p62/SQSTM1 expression and upregulated Beclin1 and ATG5 expression in a dose-dependent manner in HCC cells (**Figure 8A**). To further explore whether CPT induces cell autophagy, we analyzed CPT-induced LC3-II/LC3-I expression, by co-culturing CPT-treated cells and autophagy inhibitors. The combined treatment of CPT and 3-MA reduced LC3-II conversion. Conversely, the combined treatment of CPT and CQ resulted in an accumulation of LC3-II (**Figure 8B**). Following treatment with CPT, TEM revealed that autophagosomes/autolysosomes were significantly accumulated in Huh7 and MHCC97-H cells (**Figure 8C**). As shown in **Figure 8D**, immunofluorescence revealed that LC3 puncta formation was increased upon CPT treatment in Huh7 and MHCC97-H cells. Taken together, these results reveal that CPT induces autophagy in Huh7 and MHCC97-H cells *in vitro*.

### Inhibition of CPT-Induced Autophagy Promotes Cell Apoptosis in Huh7 and MHCC97-H Cells

**Figure 9:**
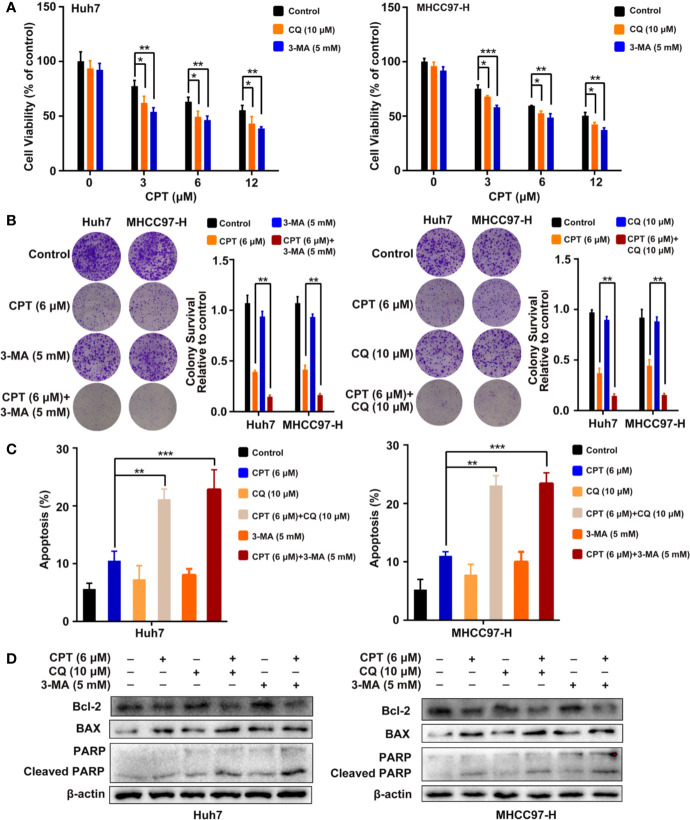
Inhibition of cryptotanshinone (CPT)-induced autophagy promotes cell apoptosis in Huh7 and MHCC97-H cells. **(A)** Huh7 and MHCC97-H cells were treated with different concentrations of CPT alone or in combination with 10 μM chloroquine (CQ) or 5 mM 3-methylamphetamine (3-MA) for 24 h. Cell viability was detected by 3-(4,5-dimethylthiazol-2-yl)-2,5-diphenyltetrazolium bromide (MTT) assay. *P < 0.05, **P < 0.01, and ***P < 0.001 versus the control group. **(B)** Huh7 and MHCC97-H cells were treated with 6 μM CPT alone or in combination with 10 μM CQ or 5 mM 3-MA for 24 h. Proliferative capacity was measured using a colony formation assay. **P < 0.01 versus the CPT group. **(C)** Flow cytometric analysis of apoptosis in Huh7 and MHCC97-H cells exposed to 6 μM CPT alone or in combination with autophagy inhibitors (3-MA or CQ) for 24 h. **P < 0.01, ***P < 0.001 versus the CPT group. **(D)** Western blot analysis of PARP, cleaved-PARP, Bcl-2, and Bax in human hepatocellular carcinoma (HCC) cells treated with 6 μM CPT alone or in combination with autophagy inhibitors (3-MA or CQ) for 24 h.

To investigate whether CPT-induced autophagy protects HCC cells, we evaluated cell proliferation and apoptosis in the presence of autophagy inhibitors (3-MA or CQ). MTT assay revealed that autophagy inhibitors significantly suppressed Huh7 and MHCC97-H cell viability when combined with CPT (**Figure 9A**). As shown in **Figure 9B**, colony formation assay demonstrated that autophagy inhibition by CQ or 3-MA inhibited HCC cell proliferation and enhanced CPT-induced apoptosis. Moreover, the annexin V-FITC apoptosis detection kit and western blot analysis were used to detect apoptosis with or without autophagy inhibitors (3-MA or CQ). As shown in **Figure 9C**, combined treatment with 3-MA or CQ and CPT caused clear effect on apoptosis in Huh7 and MHCC97-H cells. In addition, western blot analysis revealed that CPT combined with 3-MA or CQ downregulated Bcl-2 expression and upregulated the expression of cleaved PARP and Bax (**Figure 9D**). Collectively, these findings suggest that suppression of CPT-mediated autophagy promotes apoptosis in Huh7 and MHCC97-H cells.

### PI3K/AKT/mTOR Signaling Is Involved in CPT-Induced Apoptosis and Autophagy

**Figure 10:**
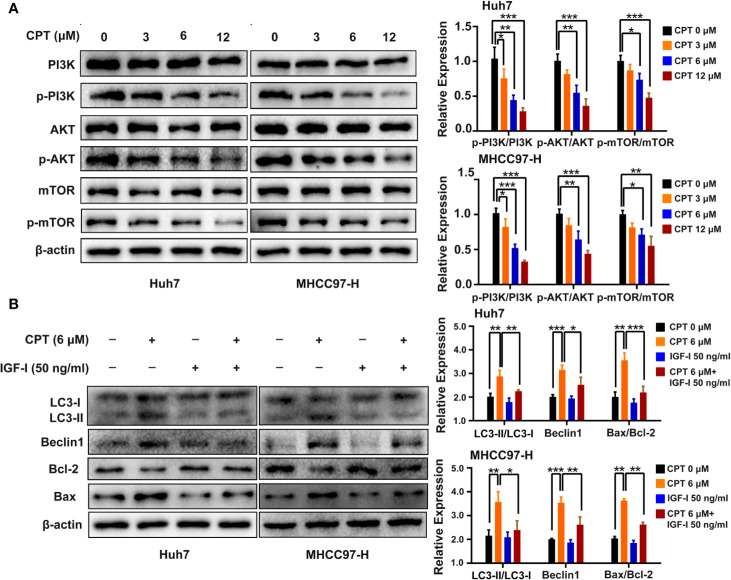
Phosphatidylinositol 3-kinase (PI3K)/protein kinase B (Akt)/mammalian target of rapamycin (mTOR) signaling pathway is involved in cryptotanshinone (CPT)-induced autophagy and apoptosis. **(A)** Western blot analysis of PI3K, p-PI3K, AKT, p-AKT, mTOR, and p-mTOR in human hepatocellular carcinoma (HCC) cells treated with different doses of CPT (0, 3, 6, and 12 μM) for 24 h. *P < 0.05, **P < 0.01, and ***P < 0.001 versus the control group. **(B)** HCC cells were exposed to 6 μM CPT alone or in combination with 50 ng/ml insulin-like growth factor-I (IGF-I). The expression levels of LC3-I, LC3-II, Beclin1, Bcl-2, and Bax proteins were assessed by western blotting.

PI3K/AKT/mTOR signaling pathway is closely related to apoptosis and autophagy. Therefore, we detected the protein expression levels of PI3K/AKT/mTOR signaling protein markers, including PI3K, p-PI3K, AKT, p-AKT, mTOR, and p-mTOR. Huh7 and MHCC97-H cells were treated with CPT, as indicated. Western blot analysis revealed that CPT decreased the expression of p-PI3K, p-AKT, and p-mTOR, whereas the expression of PI3K, AKT, and mTOR was not significantly changed (**Figure 10A**). These results indicate that CPT inhibits PI3K/AKT/mTOR signaling pathway in Huh7 and MHCC97-H cells. To further investigate the role of PI3K/AKT/mTOR signaling pathway in CPT-induced autophagy and apoptosis, we treated cells with the PI3K activator IGF-I. As shown in **Figure 10B**, CPT combined with IGF-I increased Bcl-2 expression and decreased Bax expression. In addition, the combined treatment of CPT and IGF-I decreased the LC3-II conversion and Beclin1 expression. Together, these results indicate that PI3K/AKT/mTOR signaling is a crucial pathway for CPT-induced apoptosis and autophagy in HCC.

### CPT Inhibits the Growth of Huh7 Cell Xenografts in Nude Mice

**Figure 11:**
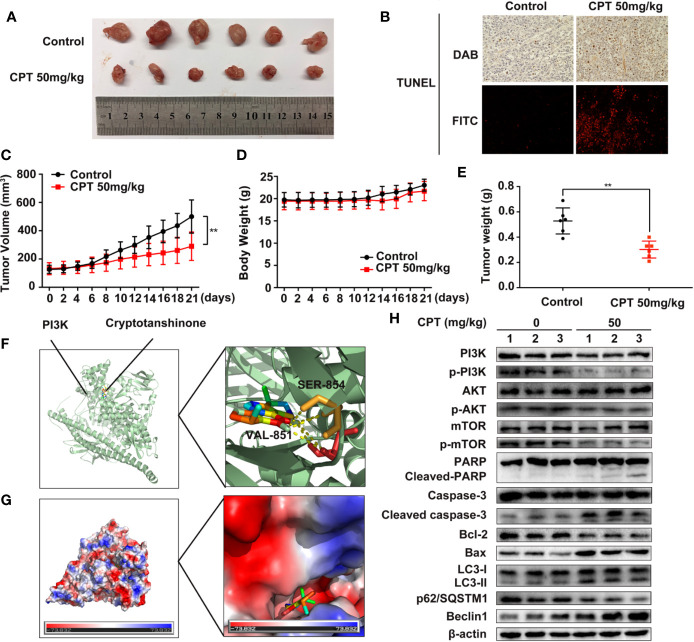
Cryptotanshinone (CPT) inhibits the growth of Huh7 cell xenografts in nude mice. **(A)** Photograph of extracted tumors from each group of mice (n = 6). Statistical analysis of tumor volumes **(C)**, body weight **(D)**, and tumor weight **(E)** in each group of mice (n = 6). **(B)** TUNEL assays were conducted to measure apoptosis in tumor tissues using immunochemistry and immunofluorescence analyses. **(F)** The binding mode of CPT with phosphatidylinositol 3-kinase (PI3K) determined by molecular docking simulation. CPT formed stable hydrogen bonds with PI3K at SER-854 and VAL-851. **(G)** Electrostatic surfaces of CPT and PI3K obtained using Pymol. Lower electrostatic potential denotes better binding ability. **(H)** Tumor tissue lysates were extracted from the control and CPT-treated groups and the expression levels of PI3K, p-PI3K, AKT, p-AKT, mTOR, p-mTOR, PARP, cleaved PARP, caspase-3, cleaved caspase-3, Bcl-2, Bax, LC3-I, LC3-II, p62/SQSTM1, and Beclin1 proteins were assessed by western blotting.

To explore the *in vivo* anti-HCC effect of CPT, the axillae of nude mice were subcutaneously injected with Huh7 cells. As shown in **Figure 11A**, CPT significantly decreased tumor growth in the CPT-treated group as compared to that of the control group. Moreover, the weight and volume of tumors were significantly lowered in the CPT-treated group as compared to those of the control group (**Figures 11C, E**). There was no significant difference in body weight between the control group and the CPT-treated group (**Figure 11D**). Immunohistochemistry and immunofluorescence analyses were performed on tissue sections from control and CPT-treated mice to detect apoptosis with TUNEL assays. Results indicate that CPT induces apoptosis in tumor tissues (**Figure 11B**). To explore how CPT affects PI3K, we assessed the potential interaction between CPT and PI3K through molecular modeling and docking simulation. This suggested a strong potential interaction between CPT and PI3K, including hydrogen bonds involving SER-854 and VAL-851 (**Figure 11F**), as well as the hydrophobic pocket comprising Tyr836, Ile932, Ile800, Glu849, Ile848, Met922, Trp780, and Gln859 (**Figure 11G**). Meanwhile, western blot analysis of tumor tissues revealed that the expression levels of p-PI3K, p-AKT, and p-mTOR were decreased in CPT-treated mice compared with that of the control group, whereas the expression of PI3K, AKT, and mTOR was not significantly changed. Markers of apoptosis, involving cleaved-PARP, cleaved caspase-3, and Bax, were increased in tumor cells, whereas of Bcl-2 was decreased. In addition, western blot analysis of tumor tissues from mouse xenografts demonstrated an increase in LC3-II conversion and Beclin 1 expression in CPT-treated mice, whereas p62/SQSTM1 expression was decreased (**Figure 11H**). These data demonstrate that CPT inhibits tumor growth in Huh7 cells, induces apoptosis and autophagy, and suppresses PI3K/AKT/mTOR signaling pathway *in vivo*.

## Discussion

Systemic therapy to treat HCC has progressed considerably since the survival benefit of sorafenib treatment was confirmed. To some extent, sorafenib increased the survival rate of patients with advanced-stage HCC, making long-term survival possible for those patients. However, patients with HCC who are administered with sorafenib undergo adverse side effects (Kudo, 2019). Hence, there is an urgent need for effective novel drugs to treat HCC with fewer side effects. CPT, a natural product derived from Radix *Salviae miltiorrhizae*, has been widely used to treat multiple cancers, including HCC. Recently, CPT has been considered a promising anticancer drug due to its obvious inhibitory effect on tumor growth. However, the mechanisms behind HCC treatment with CPT are still elusive. In this study, we utilized a systems pharmacology approach to elucidate the pharmacological mechanism of CPT in the treatment of HCC. Further experimental verification reveal that CPT has a potential antitumor effect on HCC, both *in vitro* and *in vivo*. CPT inhibited HCC growth by inhibiting cell proliferation and inducing apoptosis and autophagy, which were involved in inhibiting PI3K/AKT/mTOR signaling pathway. Autophagy inhibition with CQ or 3-MA enhanced the antitumor effect of CPT by inhibiting cell proliferation and inducing apoptosis. Interestingly, treatment with IGF-I, an activator of PI3K, simultaneously inhibited CPT-induced apoptosis and autophagy. These findings indicate that CPT inhibits proliferation and induces apoptosis and autophagy* via* the PI3K/AKT/mTOR signaling pathway, ultimately inhibiting tumor growth in HCC cells.

Caspases, the initiators and executioners of apoptosis, are the central mechanisms of apoptosis and can activate two main pathways: the intrinsic mitochondrial pathway and the extrinsic death receptor pathway (Jan and Chaudhry, 2019; Van Opdenbosch and Lamkanfi, 2019). Caspase-9 is the upstream caspase in the intrinsic pathway, whereas caspase-8 is the upstream caspase in the extrinsic pathway (Wong, 2011). The intrinsic and extrinsic pathways converge on downstream caspases such as caspase-3, caspase-6, and caspase-7, which lead to the cleavage of nuclear DNA, nuclear proteins, and cytoplasmic proteins, resulting in cell collapse and death (Ashkenazi, 2015). The Bcl-2 family of proteins consists of pro-apoptotic proteins and anti-apoptotic proteins that play critical roles in regulating apoptosis *via* the intrinsic mitochondrial pathway (Liu et al., 2006). Bcl-2 family members were further divided into three groups based on their functions and Bcl-2 homology domains. The first group is composed of anti-apoptotic proteins containing all four BH domains, including Bcl-2, Bcl-xL, Bcl-B/Bcl2L, Bcl-w, Mcl-1, and A1/Bfl-1. The second group consists of pro-apoptotic BH-3 proteins, including Bid, Bim, Noxa, Puma, Bmf, Bad, Bik, and Hrk. All pro-apoptotic proteins containing all four BH domains, such as Bax, Bok/Mtd, and Bak, form the third group (Kale et al., 2018). In this study, target-function network analysis indicated that the therapeutic mechanism of CPT in HCC treatment involved various targets that regulate cell apoptosis. In addition, we revealed that Bcl-2 expression was significantly decreased, whereas Bax, p-PARP, and cleaved caspase-3 expression was increased following CPT treatment. Taken together, these results indicate that CPT treatment induces apoptosis in Huh7 and MHCC97-H cells. Considering the role of autophagy in cancer pathogenesis, we investigated whether CPT can modulate the autophagic signaling cascade. Autophagy is a degrading process in which autophagosomes package damaged organelles or cellular debris fused with lysosomes for degradation (Levy et al., 2017; Towers et al., 2019). LC3, a widely used marker, plays a crucial role in autophagosome biogenesis and autophagy substrate selection (Shi et al., 2017). The selective substrate of autophagy, p62/SQSTM1, is degraded during autophagy. Meanwhile, autophagy markers such as ATG5 and Beclin1 are closely related to autophagosomes (Lamark et al., 2017). In this study, as shown in **Figure 4**, target-function network analysis revealed that the mechanism of CPT action in HCC treatment involved various targets regulating cell autophagy. Furthermore, we investigated several autophagy-related proteins, including LC3-I/LC3-II, p62/SQSTM1, Beclin1, and ATG5. LC3-II conversion and the expression levels of Beclin1 and ATG5 were increased by CPT treatment, and only p62/SQSTM1 expression was reduced. Moreover, autophagosomes/autolysosomes were significantly increased in Huh7 and MHCC97-H cells treated with CPT, as revealed by TEM and immunofluorescence analysis. Thus, CPT induced autophagy in Huh7 and MHCC97-H cells. However, whether autophagy promotes or inhibits tumor growth remains controversial. In the present study, using the autophagy inhibitors, 3-MA and CQ, we further explored the role of autophagy in CPT-induced apoptosis and proliferation inhibition. CPT decreased Bcl-2 levels and increased p-PARP and Bax levels; subsequently, the increased levels of apoptosis were further enhanced by 3-MA and CQ. Furthermore, treatment with CPT in combination with autophagy inhibitors (3-MA or CQ) led to an enhanced inhibition of cell viability. Therefore, we found that inhibiting autophagy could enhance CPT-induced apoptosis, indicating that autophagy played a potential defensive mechanism against HCC treatment agents in Huh7 and MHCC97-H cells.

**Figure 12:**
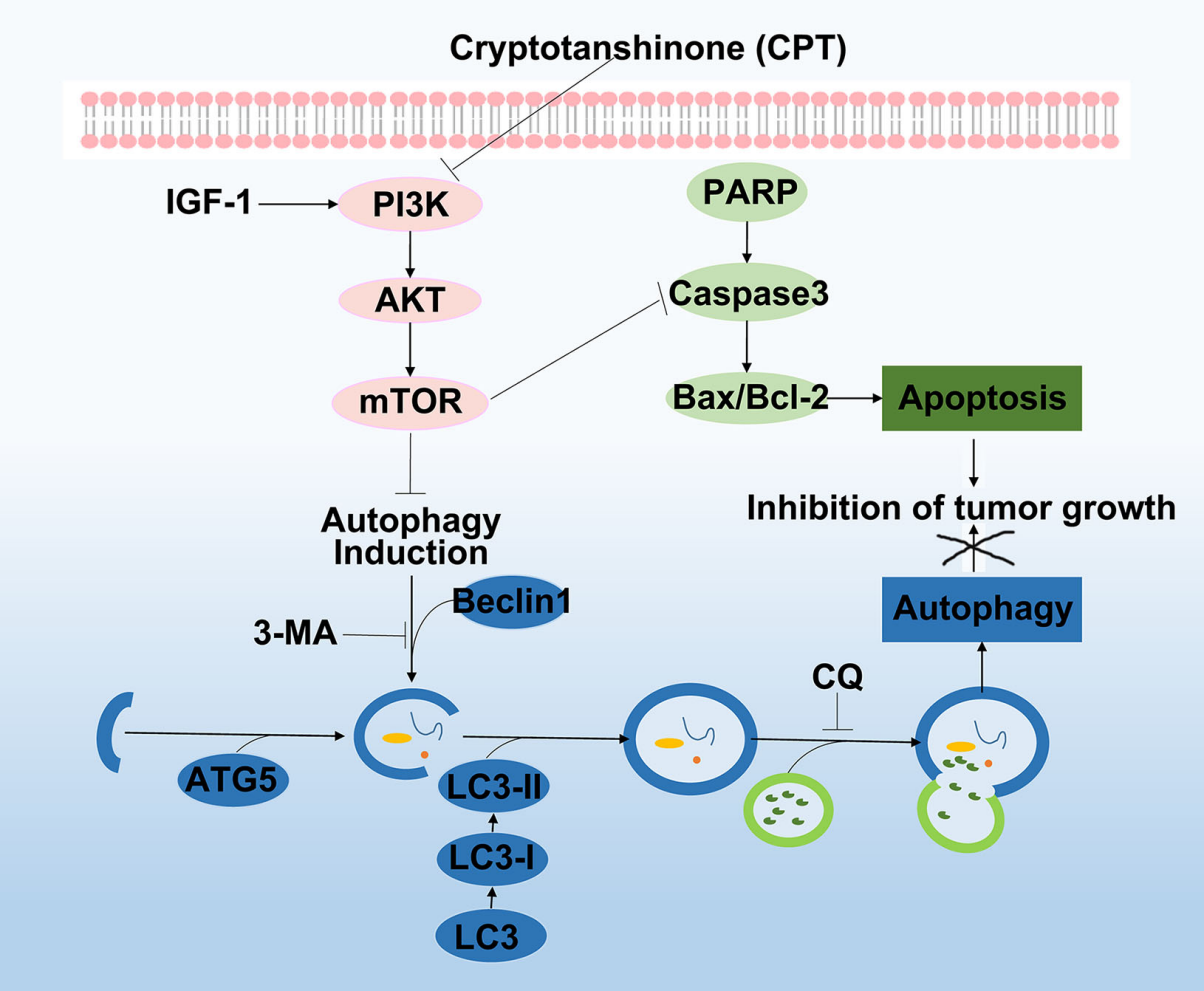
Working model of the experimental validation. Diagram indicating cryptotanshinone (CPT)-induced autophagy and apoptosis in human hepatocellular carcinoma (HCC) *via* inhibition of the phosphatidylinositol 3-kinase (PI3K)/protein kinase B (Akt)/mammalian target of rapamycin (mTOR) signaling pathway.

PI3K/Akt/mTOR signaling pathway is one of the most significant signaling pathways, playing a critical role in essential intracellular functions (Emerling and Akcakanat, 2011; Janku et al., 2018). Activation of PI3K activates signal transduction pathways that promote cancer cell growth, survival, and metabolism (Alzahrani, 2019). AKT, an essential downstream effector of PI3K during tumorigenesis, is a serine-threonine kinase directly activated by PI3K (Wu and Hu, 2010). mTOR, a critical downstream modulator of AKT, is composed of two structurally unique complexes, namely, mTOR complex 1 (mTORC1) and mTOR complex 2 (mTORC2), which are regulated by multiple pathways (Guertin and Sabatini, 2007). Previous studies have suggested that PI3K/AKT/mTOR signaling pathway is closely associated with autophagy and functions to regulate tumor cell proliferation and apoptosis (Sun et al., 2013; Yoshida, 2017). It was reported that arenobufagin induces apoptosis and autophagy in HCC cells by suppressing the PI3K/AKT/mTOR pathway (Zhang et al., 2013). Further, apoptosis is induced by caffeine primarily through the enhancement of autophagy *via* inhibition of PI3K/Akt/mTOR/p70S6K signaling pathway (Saiki et al., 2011). In our study, as discussed in **Figure 5**, pathway enrichment analysis indicated that targeting the PI3K/Akt/mTOR signaling pathway provides a potential therapeutic insight for HCC treatment. Moreover, we found that CPT significantly inhibited PI3K/AKT/mTOR signaling pathway by sequential reduction of downstream proteins, including p-PI3K, p-AKT, and p-mTOR. In addition, treatment with the PI3K activator IGF-I in combination with CPT inhibited CPT-induced cell apoptosis and autophagy. Moreover, CPT formed stable hydrogen bonds with PI3K at SER-854 and VAL-851. Here, we demonstrated that suppression of the PI3K/AKT/mTOR signaling pathway is a crucial factor in regulating CPT-induced apoptosis and autophagy (**Figure 12**).

However, there are some limitations to this study. First, we predicted multiple targets, biological functions, and pathways through a systems pharmacology approach. Due to time and monetary constraints, we selected only targets that we were interested in and did not validate all targets one by one. In addition, this study explored the relationship between apoptosis and autophagy *in vitro*, but not *in vivo*; therefore, further animal or clinical studies are needed to investigate this. Nevertheless, this study provides powerful support for further systematic and in-depth study of the anti-HCC mechanism of CPT in the future. CPT may be a potential novel anti-HCC drug.

## Conclusions

In conclusion, the pharmacological mechanisms of CPT action against HCC were explored through a combination of network pharmacology analysis and experimental validation. We demonstrated that CPT inhibited proliferation and induced apoptosis and autophagy in Huh7 and MHCC97-H cells through PI3K/AKT/mTOR-mediated pathways. We also found that inhibition of CPT-induced autophagy enhanced the antiproliferative and apoptosis effects in Huh7 and MHCC97-H cells. In addition, PI3K activation inhibited CPT-induced apoptosis. These findings provide new insights into the molecular mechanisms of HCC pathogenesis and implicate the PI3K/AKT/mTOR signaling pathway as a potential therapeutic target in HCC treatment.

## Data Availability Statement

All datasets presented in this study are included in the article/**Supplementary Material**.

## Ethics Statement

The animal study was reviewed and approved by Animal Ethics Committee of Guangzhou University of Chinese Medicine.

## Author Contributions

MH and ZY designed the study. YLu and LS did *in vitro* and *in vivo* experiments. XW analyzed the data. YLu wrote the manuscript. YH and YLi revised the final manuscript. QW, MH, and ZY provided advice during the study and manuscript preparation.

## Funding

This study was supported by grants from Guangzhou Science Technology and Innovation Commission Technology Research Projects (No. 201805010005) to QW. Funders provided financial support for the study.

## Conflict of Interest

The authors declare that the research was conducted in the absence of any commercial or financial relationships that could be construed as a potential conflict of interest.

## Supplementary Material

Table S1 Targets of CPT used for network construction.

Table S2 List of HCC-related targets.

Table S3 List of the common targets of CPT and HCC.

Table S4 List of the 32 hub targets in this PPI network.

Table S5 Functional classification of the common targets based on DAVID analysis.

Table S6 Pathway enrichment results based on the common targets of CPT and HCC.
